# Health-related quality of life associated with different cervical cancer therapies received by patients in two Nigerian tertiary hospitals

**DOI:** 10.4314/ahs.v23i3.32

**Published:** 2023-09

**Authors:** Aliyu Samaila, Aminu A Biambo, Nuruddeen Usman, Usman M Aliyu, Adamu Abdullahi, Maxwell O Adibe

**Affiliations:** 1 Usmanu Danfodiyo University Sokoto; 2 Usmanu Danfodiyo University Teaching Hospital, Sokoto; 3 Ahmadu Bello University Teaching Hospital, Zaria; 4 University of Nigeria Nsukka

**Keywords:** Cervical cancer, quality of life, therapies

## Abstract

**Background:**

There are important consequences from cervical cancer (CC) disease and its treatment among survivors, especially the impact on quality of life (QoL).

**Objective:**

To evaluate the health-related QoL associated with different CC therapies received by patients in two Nigerian tertiary hospitals.

**Methods:**

This study employed a prospective longitudinal design. It was conducted at Usmanu Danfodiyo University Teaching Hospital, Sokoto and Ahmadu Bello University Teaching Hospital Zaria, North-Western Nigeria. Data of all the 157 eligible CC patients were collected at baseline and after therapy. Data analysis was done with appropriate descriptive and inferential statistics using SPSS V. 20 for windows. *P*<0.05 was considered statistically significant.

**Results:**

Chemotherapy (CT) was the major therapy option received by 78(49.7%) of the patients. Patients who received chemoradiation therapy (CRT) and adjuvant chemotherapy (CTS) had the highest increase in mean overall health-related QoL of 0.138 (*t*=8.456, p<0.001) and 0.138 (*t*=6.489, *p*<0.001) higher than their respective baseline scores. Patients who received CT had the least increase in mean overall health-related QoL of 0.095 (*t*=4.574, *p*<0.001) from baseline.

**Conclusion:**

Chemoradiation therapy and adjuvant chemotherapy were associated with highest increase in mean overall health-related QoL. Chemotherapy was associated with the least increase in mean overall health-related QoL.

## Introduction

Cervical cancer (CC) is the most common gynecological malignancy 1,2, and the fourth most common cancer among women worldwide. CC is still a major cause of morbidity and mortality among women in resource-poor regions of Africa. 3. According to the 2020 report of the Global Cancer Observatory (GLOBOCAN), there were an estimated 604,127 new cases worldwide with 27, 806 and 12, 075 cases from West Africa and Nigeria, respectively^4^.

Treatment options for CC, according to the National Comprehensive Cancer Network (NCCN) Guidelines, include surgery (cervical cone biopsy, radical hysterectomy, radical trachelectomy, etc.), radiotherapy (pelvic external beam radiation therapy and intracavitary brachytherapy), cisplatin-based concurrent chemoradiation, cisplatin or non-cisplatin-based chemotherapy with or without surgery. 5. Despite the fact that CC has a diagnosis and therapy, the disease and its treatment have significant repercussions for survivors, particularly in terms of QoL. Some functional disorders occur following therapies such as surgery, which involves the female genital anatomy directly affect their perception of body image and sexual functions. Radiotherapy could damage the vaginal mucosa and epithelium; chemotherapy could induce various adverse effects like nausea, vomiting, diarrhea, constipation, mucositis, weight changes and hormonal changes. Various psychological factors including low self-esteem, changes in self-image, beliefs about the origin of cancer, marital tensions, fears and worries can affect the patients. In addition, the survivors believed they were sick and were not contributing members of their communities 6,7. However, studies have shown that optimal treatment of patients improves overall QoLs 6–13.

In most African countries, the resources required for effective CC treatment are scarce. In Nigeria, for example, treatment services for premalignant and malignant lesions are woefully inadequate. Radiotherapy is accessible in ten locations around the country, with two machines operating on average at any given time. 14. There are few studies on the quality of life (QoL) of cervical cancer survivors (CCS) treated with the various therapy options in our health-care institutions. 15. Evidence is necessary of patients' self-evaluation of their therapies and overall perception of care because we live in an era of evidence-based health care. For the above-mentioned reasons, this present study was carried out to evaluate the health-related quality of life associated with different CC therapies received by patients in two Nigerian tertiary hospitals.

## Methods

### Study design and settings

This study employed a prospective longitudinal design. It was conducted at Usmanu Danfodiyo University Teaching Hospital (UDUTH), Sokoto in Sokoto state and Ahmadu Bello University Teaching Hospital (ABUTH), Zaria in Kaduna state, North-Western Nigeria.

### Study participants and eligibility requirements

The study included patients with CC who planned to be treated with chemotherapy, radiation, or both, with or without surgery.

Patients who were just going to be treated with surgery were not included in the study.

Patients who began therapy and were referred from facilities other than ABUTH or UDUTH and for whom baseline information could not be acquired were excluded from the research.

Patients who were unable to answer to interviews due to their illness' severity and those who refused to consent to participate in the study were also excluded.

### Sampling technique

ABUTH and UDUTH were purposively sampled as the study settings being the major cancer registries in the North-Western Nigeria. During the six-month recruitment period (January - June, 2019), all eligible patients who visited the hospitals' Radiotherapy and Oncology clinics were recruited. (January - June, 2019).

## Study instruments

### The Pro Forma

This consisted of four (4) sections (A-D). Section A: consist of the patient's socio-demographic information, B: base-line clinical profile of the patient, C: treatment(s) received and D: follow-ups.

### A Generic 15D© Quality of Life Questionnaire

The 15D is a generic, comprehensive, 15-dimensional, standardized, self-administered measure of health-related quality of life (HRQoL) that can be used both as a profile and single index score measure 16. The instrument was used to collect data of the patients' QoLs before and after therapy. The 15-domains include: Mobility, Vision, Hearing, Breathing, Sleeping, Eating, Speech, Excretion, Usual Activities, Mental Function, Discomfort and Symptoms, Depression, Distress, Vitality and Sexual Activity domain. Each domain has five question items (1 to 5) with the first question item indicating the best, while the last indicating the worst QoL.

### Study procedure

Patients' recruitment was opened for a period of six (6) months (from January to June, 2019) at the Radiotherapy and Oncology Departments of the hospitals. Each recruited patient was followed-up for a period of 12 months. The complete study period was 18 months (January, 2019-June, 2020). Research assistants were recruited and trained using all relevant materials such as the study instruments, sample patient's folder and the radiation cards, to ensure quality data collection. The QoLs data of the patients were collected via self-administration of the 15D QoL instrument at baseline and after the patient completed or due to have completed her prescribed therapy option. Patients with difficulties were assisted with due consideration to the standard protocol for questionnaire administration to reduce potential bias. Data collected on the patients' therapy option received include the cytotoxic drugs received, details of radiation exposure and CC surgical interventions.

### Ethical Considerations

Ethical approvals were obtained from the Health Research Ethics Committees of UDUTH (UDUTH/HREC/2018/No.731), and ABUTH (ABUTH/HREC/CL/05) before the commencement of the data collection. The confidentiality and anonymity of the patients were maintained during and after the study.

### Data analysis

The data obtained were sorted, coded and entered into SPSS package V. 20 (SPSS Inc., Chicago, IL, USA) for windows and subsequently analysed. The data were summarized as frequencies, percentages and means ± SD. Pearson's Chi square was used to determine the associations between the therapy options received and categorized QoL scores at baseline and after therapy. Paired t-test was used to test for the mean difference in QoL associated with different therapy options before and after therapy. The overall QoL scores before and after therapy were categorized as: Poor (≤0.20), Fair (0.20-0.40), Good (0.40-0.60), Better (0.60-0.80) and Best QoL (0.80-1.00). This categorization was based on the nature of the 15D questions items and measurement scales. A priori significance level of *p<* 0.05 was used throughout.

## Results

During the six-month recruiting phase, a total of 205 patients were recruited from the hospitals.

Six patients failed to meet the qualifying criteria, eight patients refused to participate in the study, and 34 patients were excluded from the study owing to a lack of follow-up. The final analysis included 157 patients.

### Socio-demographic and clinical characteristics of the patients

The mean age of the patients was 50.7±9.0 years. Most of the patients 68(43.3%), earned an average of <N50,000.00 (<$139) per month. Majority of the patients 74(47.1%) presented with baseline clinical stage III, while stage IVB 5(3.2%), (*p*<0.001), was the least presented clinical stage at baseline. Squamous Cell Carcinoma 144(92.3%) was the major histological type. Other details of the patients' baseline socio-demographic and characteristics of the patients can be seen in Table 1 below.

**Table 1:** Socio-demographic and clinical characteristics of the patients

Demographic & Clinical Data	(N = 157)	n (%)
Mean Age (Years)	**50.7±9.0**	
Mean BSA (m^2^)		1.63±0.18
Marital Status		
Single		1(0.6)
Married		128(81.5)
Divorced		12(7.6)
Widow		16(10.2)
Parity		
Nulliparous		1(0.6)
Uniparous		2(1.3)
Multiparous		154(98.1)
Level of Education		
Non-Formal		61(38.9)
Primary		9(5.7)
Secondary		54(34.4)
Tertiary		33(21.0)
Occupation		
Unemployed		9(5.7)
House Wife		92(58.5)
Business		24(15.3)
Civil servant		26(16.6)
Farmer		3(1.9)
Student		3(1.9)
Average Monthly Income		
**<**₦50,000 ($139)		68(43.3)
₦50,000-100,000 ($139-278)		66(42.0)
**>**₦100,000 (>$278)		23(14.6)
Clinical Stage		
I		8(5.1)
II		59(37.6)
III		74(47.1)
IVA		11(7.0)
IVB		5(3.2)
Histological Type (n=*156*)		
Squamous Cell Carcinoma		144(92.3)
Adenocarcinoma		12(7.7)

$1=₦360, using 2019 exchange rate

### Therapy options received by the patients

A total of six therapy options including chemotherapy (CT), radiation therapy (RT), chemoradiation therapy (CRT), adjuvant chemotherapy (CTS), adjuvant radiation therapy (RTS) and adjuvant chemoradiation therapy (CRTS) were used among the 157 CC patients. A total of 78(49.7%) patients were placed on CT, making it the main therapy option received by the patients, while 1(0.6%) was prescribed RT making it the least therapy option received by the patients. CRT, CTS, RTS, and CRTS were received by 51(32.5%), 7(4.5%), 4(2.5%) and 16(10.2%), *p*<0.001 patients respectively. External beam radiation therapy (EBRT) was the only form of radiation received by patients treated with radiation therapy.

### Health-related quality of lives associated with different therapy options received by the patients

None of the patients who received any of the therapy options reported ‘Poor’ or ‘Fair’ QoL before and after the therapy. Before commencing therapy, patients who received CT, 17(21.8%), CRT, 11(21.6%), RTS, 4(100.0%) and CRTS, 2(12.5%), *p*<0.001 reported the ‘Best’ QoL. After therapy, patients who received CT, 48(61.5%), CRT, 45(88.2%), RTS, 4(100.0%), CRTS, 15(93.8%), and CTS, 7(100.0%), p=0.002 reported the ‘Best’ QoL. There was significant association between therapy option received and the patients' reported health-related QoL after therapy at *p*<0.05. Details can be seen in Table 2 below.

**Table 2:** Associations between the therapy options received and categorized QoL scores at baseline and after therapy

Categorized QoL	Therapy options received (N=157)						

	CT	RT	CRT	CTS	RTS	CRTS	*P-Value*
Baseline QoL							
Poor, n (%)	-	-	-	-	-	-	
Fair, n (%)	-	-	-	-	-	-	
Good, n (%)	14(17.9)	1(100.0)	4(7.8)	0(0.0)	0(0.0)	0(0.0)	<0.001
Better, n (%)	47(60.3)	0(0.0)	36(70.6)	7(100.0)	0(0.0)	14(87.5)	
Best, n (%)	17(21.8)	0(0.0)	11(21.6)	0(0.0)	4(100.0)	2(12.5)	
							
QoL after therapy							
Poor, n (%)	-	-	-	-	-	-	
Fair, n (%)	-	-	-	-	-	-	
Good, n (%)	12(15.4)	1(100.0)	3(5.9)	0(0.0)	0(0.0)	1(6.3)	0.002
Better, n (%)	18(23.1)	0(0.0)	3(5.9)	0(0.0)	0(0.0)	0(0.0)	
Best, n (%)	48(61.5)	0(0.0)	45(88.2)	7(100.0)	4(100.0)	15(93.8)	

CT=Chemotherapy, RT=Radiation Therapy, CRT=Chemoradiation Therapy, CTS=Chemotherapy and Surgery, RTS=Radiation Therapy and Surgery, CRTS= Chemoradiation Therapy and Surgery, QoL=Quality

After therapy, patients who received CRT and CTS had the highest increase in mean overall health-related QoL of 0.138 (*t*=8.456, *p*<0.001) and 0.138 (*t*=6.489, *p*<0.001) higher than their respective baseline scores. Patients who received CT had the least increase in mean overall health-related QoL of 0.095 (*t*=4.574, *p*<0.001) from baseline. Patients who received CRTS had the highest increase in single attributes QoL ‘Excretion and Discomfort/Symptoms’, 0.494±0.184 and 0.429±0.125, *p*<0.001 respectively. Details of the overall mean difference and single attributes mean differences in QoL associated with different therapy options from baseline can be seen in Table 3 below.

**Table 3:** Overall mean difference and single attributes mean difference in QoL associated with different therapy options from baseline

After Therapy-Baseline	CT		CRT		CTS		RTS		CRTS	
	MD±SD	*P-Value*	MD±SD	*P-Value*	MD±SD	*P-Value*	MD±SD	*P-Value*	MD±SD	*P-Value*
	
Overall HRQoL	0.095±0.183	<0.001	0.138±0.117	<0.001	0.138±0.056	<0.001	0.107±0.021	0.002	0.131±0.113	<0.001
Single attributes										
Mobility	0.039±0.247	0.162	0.057±0.207	0.056	-	-	-	-	0.093±0.218	0.111
Vision	- 0.049±0.213	0.043	0.011±0.175	0.650	-	-	-	-	-0.032±0.128	0.333
Hearing	-	0.010	0.004±0.181	0.870	-	-	-	-	-0.034±0.135	0.333
	0.065±0.217									
Breathing	0.034±0.238	0.204	0.019±0.199	0.491	-	-	-	-	-0.033±0.131	0.333
Sleeping	0.197±0.297	<0.001	0.257±0.155	<0.001	0.317±0.116	<0.001	0.366±0.244	0.058	0.062±0.142	0.102
Eating	0.027±0.299	0.422	- 0.011±0.181	0.659	-	-	-	-	0.008±0.196	0.866
Speech	-	0.170	0.029±0.197	0.192	-	-	-	-	-0.017±.068	0.333
	0.035±0.226									
Excretion	0.282±0.229	<0.001	0.291±0.197	<0.001	0.328±0.081	<0.001	-	-	0.494±0.184	<0.001
Usual Activities	0.146±0.326	<0.001	0.309±0.154	<0.001	0.256±0.113	0.001	0.279	-	0.297±0.108	<0.001
Mental	-	0.278	0.037±0.216	0.230	0.102±0.174	0.172	-	-	0.006±0.207	0.917
Function	0.037±0.296									
Discom. and Symp	0.285±0.229	<0.001	0.267±0.181	<0.001	0.418±0.144	<0.001	0.298	-	0.429±0.125	<0.001
Depression	0.086±0.189	<0.001	0.214±0.150	<0.001	0.126±0.177	0.110	0.176±0.117	0.058	0.125±0.129	0.002
Distress	0.141±0.227	<0.001	0.279±0.204	<0.001	0.215±0.269	0.079	0.275	-	0.178±0.207	0.004
Vitality	0.181±0.239	<0.001	0.272±0.119	<0.001	0.211±0.094	0.001	0.229	-	0.217±0.126	<0.001
Sexual Activity	0.107±0.149	<0.001	0.091±0.173	<0.001	0.028±0.073	0.356	0.218±0.145	0.058	0.101±0.202	0.064

HRQoL=Health Related Quality of Life

## Discussion

This study was conducted to evaluate the health-related quality of life associated with different CC therapies received by patients in two Nigerian tertiary hospitals. Prior research has found that the average age of the patients in our study is similar to that of previous investigations. 17–19. A rise in CC was seen with increasing age, parity, early and prolonged sexual period 18. Majority of the patients were low-income earners. A study conducted in Abuja; Nigeria revealed that the direct cost of RT for CC is N600,000 per course of teletherapy plus approximately N150,000 for pre-treatment evaluation in the FCT 14. This demonstrated how the majority of CC patients in Nigeria face the greatest difficulty of affordability. In our study, majority of the patients presented with advanced-stage disease. This is in line with earlier research done in Nigeria. 20,21. In contrast, a study showed that, in United Kingdom, only 21.9% of women present with the advanced-stage disease 22. Squamous cell carcinoma, large cell non-keratinizing (SCCLCNK) was the most presented histological type. Prior research conducted reported similar findings 2,18. CT and RT have been found to be the most common and least common treatment options for patients. A study conducted to provide comprehensive treatment given to CC patients showed that, majority (97%) of the patients received external beam radiation, 84% brachytherapy, and only 4% received concomitant chemotherapy 23. Kumar et al., reported that radical hysterectomy was the most common treatment modality followed by Wertheim's Hysterectomy and Radio-chemotherapy 24.

It can be seen that the overall QoLs of the patients improved after therapy. Similar studies on QoLs of CC patients before and after therapy also reported improvement in QoLs after therapy, even though these studies used different instruments 6,11,12. Patients who received CRT, CTS had the highest increase in mean overall HRQoL compared to those patients who received CT. The single attributes “discomfort/symptoms and excretion” were seen to have the highest increase in mean QoLs scores in patients who received CRTS. “Usual activities and excretion” in patients who received CRT had the highest increase in mean QoL scores after therapy. Patients who received RTS were seen to have achieved better increase in sexual activity attribute of QoL score after therapy. This might be due to the fact that, all patients who received RTS belong to clinical stage I with less disease burden. This can be supported by a study which indicated that patients with precancerous lesions and early CC show better overall QoL than do those with advanced stage-disease. Additionally, patients with early cancer recover more quickly than do those with the advanced disease in terms of both physical and mental functions 25. A number of studies have reported that CC survivors experience decreased sexual, physical, social and emotional wellbeing 8,11,24. However, Rahman et al., reported that, although there was no significant improvement in social, cognitive, or role functioning, body image, sexual activity, or sexual enjoyment with worsened vaginal and sexual function, the QoL of the patients in terms of physical and emotional functioning improved with treatment 26.

## Conclusion

Six different therapy options including chemotherapy (CT), radiation therapy (RT), chemoradiation therapy (CRT), adjuvant chemotherapy (CTS), adjuvant radiation therapy (RTS) and adjuvant chemoradiation therapy (CRTS) were used among the patients. CRT and CTS were found to be associated with highest increase in mean overall health-related QoL. Exclusive CT was found to be associated with the least increase in mean overall health-related QoL. CRTS was found to be associated with the highest increase in single attributes QoL related to ‘Excretion and Discomfort/Symptoms’.

## Strengths of the study

The study was conducted in the major cancer registries in the study area.

The study was collaborative involving consultant clinical radio-oncologists and clinical pharmacy academics. The study used English version of the 15D QoL Questionnaire, which is highly reliable, sensitive and responsive to change.

## Limitations of the study

One of the limitations to this study is its observational nature because, the potential for bias is higher in observational studies. However, this was minimized by proper training of the research assistants on the implications of observer bias in the study.

Attrition bias or loss during follow-up was also serious threat to this study, although this was minimized by the support of the care providers in encouraging the patients' adherence to scheduled follow-up visits.
